# Differential effects of two phosphodiesterase 4 inhibitors against lipopolysaccharide-induced neuroinflammation in mice

**DOI:** 10.1186/s12868-023-00810-7

**Published:** 2023-07-31

**Authors:** Dong Ho Kang, Sunjoo Ahn, Jung Woo Chae, Jin Sook Song

**Affiliations:** 1 Data Convergence Drug Research Center, Therapeutics & Biotechnology Division, Research Institute of Chemical Technology, 141 Gajeong-ro, Yuseong-gu, Daejeon, 34114 Korea; 2 College of Pharmacy, Chungnam National University, Daejeon, Korea

**Keywords:** PDE4B, PDE4D, Neuroinflammation, Roflumilast, Zatolmilast, Brain dispostion

## Abstract

**Background:**

Several phosphodiesterase 4 (PDE4) inhibitors have emerged as potential therapeutics for central nervous system (CNS) diseases. This study investigated the pharmacological effects of two selective PDE4 inhibitors, roflumilast and zatolmilast, against lipopolysaccharide-induced neuroinflammation.

**Results:**

In BV-2 cells, the PDE4 inhibitor roflumilast reduced the production of nitric oxide and tumor necrosis factor-α (TNF-α) by inhibiting NF-κB phosphorylation. Moreover, mice administered roflumilast had significantly reduced TNF-α, interleukin-1β (IL-1β), and IL-6 levels in plasma and brain tissues. By contrast, zatolmilast, a PDE4D inhibitor, showed no anti-neuroinflammatory effects in vitro or in vivo. Next, in vitro and in vivo pharmacokinetic studies of these compounds in the brain were performed. The apparent permeability coefficients of 3 µM roflumilast and zatolmilast were high (> 23 × 10^–6^ cm/s) and moderate (3.72–7.18 × 10^–6^ cm/s), respectively, and increased in a concentration-dependent manner in the MDR1-MDCK monolayer. The efflux ratios were < 1.92, suggesting that these compounds are not P-glycoprotein substrates. Following oral administration, both roflumilast and zatolmilast were slowly absorbed and eliminated, with time-to-peak drug concentrations of 2–2.3 h and terminal half-lives of 7–20 h. Assessment of their brain dispositions revealed the unbound brain-to-plasma partition coefficients of roflumilast and zatolmilast to be 0.17 and 0.18, respectively.

**Conclusions:**

These findings suggest that roflumilast, but not zatolmilast, has the potential for use as a therapeutic agent against neuroinflammatory diseases.

**Supplementary Information:**

The online version contains supplementary material available at 10.1186/s12868-023-00810-7.

## Background

Phosphodiesterases (PDEs) are the only known enzymes capable of breaking down cyclic nucleotides such as cyclic adenosine monophosphate (cAMP) or cyclic guanosine monophosphate (cGMP), and are divided into 11 families (PDE1–11) [1, 2]. Because of their critical role in various cellular functions, they are emerging as a target of new therapeutics for inflammation [3]. Among cAMP-hydrolyzing PDEs, PDE4 accounts for most cAMP-dependent PDE activity in the central nervous system (CNS) [4]. Thus, PDE4 inhibition has been an important strategy for resolving neuroinflammation, conferring neuroprotection, and promoting cognitive function in various CNS disease models by increasing cAMP levels [5, 6]. The PDE4 family consists of four homolog genes (PDE4A–D), among which PDE4B and PDE4D are highly expressed in the brain and may be good candidate targets for drugs to treat CNS disorders [1, 5, 7].

Roflumilast, a selective PDE4 inhibitor that inhibits all PDE4 subtypes to similar extents (with a half-maximal inhibitory concentration [IC_50_] of 0.2–0.9 nM against PDE4As, PDE4Bs and PDE4Ds and 3–4.3 nM against PDE4Cs) [8]. It has anti-inflammatory effects and is approved by the Food and Drug Administration (FDA) for the treatment of severe chronic pulmonary disease [8, 9]. Moreover, in one study, roflumilast enhanced memory in an object-location task and spatial Y-maze in rodents [10]. In a study of an APP/PS1 mouse model of Alzheimer’s disease, roflumilast (0.1, 0.2, and 0.4 mg/kg) administered intragastrically at a volume of 0.1 mL/10 g for 3 weeks restored performance in the Morris water maze, novel object recognition, and passive avoidance tasks and attenuated neuronal cell injuries and apoptosis via PDE4B/4D-mediated cAMP/CREB/BDNF signaling in the hippocampus and cerebral cortex [11]. Subcutaneous administration of roflumilast (1 mL saline containing 3% DMSO, 3 mg/kg) decreased tumor necrosis factor-α (TNF-α), interleukin-1β (IL-1β), and IL-6 levels and the number of apoptotic neurons in the brain following subarachnoid hemorrhage [12].

Zatolmilast (BPN14770), a selective allosteric PDE4D inhibitor, which binds to a primate-specific, N-terminal region with IC_50_ values of 7.8 nM and 7.4 nM against PDE4D7 and PDE4D3, respectively [13]. It is in a phase 3 clinical study as a therapeutic agent for fragile X syndrome, a rare genetic disorder [14, 15]. It increases brain cAMP levels and the phosphorylation of cAMP response element-binding protein (CREB) and brain-derived neurotrophic factor, markers of memory consolidation. It can also enhance memory and cognitive function and protect neurons, and is a potential clinical treatment for Alzheimer’s disease [16, 17].

Microglia and macrophages are widely distributed in the brain and have vital roles in innate immune responses and neural homeostasis [5, 18]. Under normal brain conditions, microglia remove damaged neurons and cell debris; in their resting phenotypes, they perform various physical functions to support neurogenesis, release neurotrophic factors, and regulate brain development [19–21]. However, abnormal activation of microglia alters their cell morphology and functionality, causing chronic neuroinflammation that in turn sustains glial cell activation via the production of various inflammatory mediators and cytokines, including nitric oxide (NO), TNF-α, IL-1β, and IL-6 [19, 22]. Such neuroinflammatory states are highly correlated with various CNS diseases, including Parkinson’s disease, Alzheimer’s disease, and Huntington’s disease. Therefore, inflammatory mediator and cytokine reduction is a potential therapeutic strategy for neuroinflammatory diseases [23, 24].

In this study, we examined the pharmacological effects of roflumilast and zatolmilast on lipopolysaccharide (LPS)-induced neuroinflammation in vitro and in vivo, and evaluated their pharmacokinetics in the brain.

## Results

### Effects of various concentrations of roflumilast and zatolmilast on NO and inflammatory cytokine production in LPS-exposed BV-2 cells

**Fig. 1:**
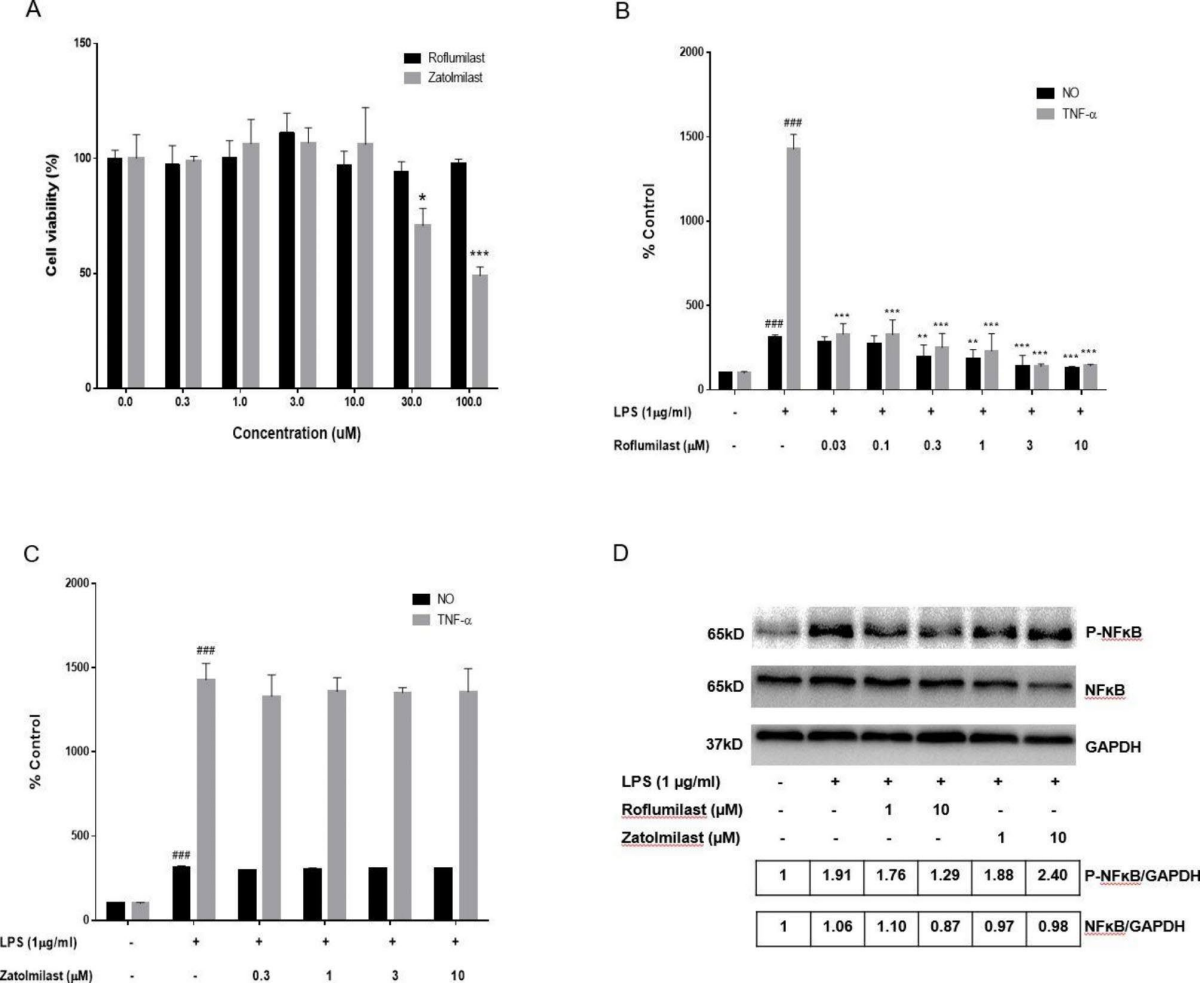
Effects of roflumilast and zatolmilast on nitric oxide (NO) and tumor necrosis factor-α (TNF-α) production in lipopolysaccharide (LPS)-induced BV-2 cells. (**A**) Roflumilast and zatolmilast cytotoxicity in BV-2 cells was assessed at concentrations up to 100 µM using the WST-8 assay. (**B, C**) NO and TNF-α production after 1 h pretreatment with roflumilast or zatolmilast with or without LPS (1 µg/mL) exposure for an additional 24 h. Culture supernatant was collected and analyzed for NO and TNF-α levels (n = 6 per group). NO levels were measured using the Griess test, and TNF-α levels were detected via enzyme-linked immunosorbent assay. The NO and TNF-α levels are presented as a percentage of untreated control cell levels. The data are expressed as the mean ± standard deviation. ^*###*^*P* < 0.001 versus the control group; ^**^P < 0.01, ^***^P < 0.001 versus the LPS group. (**D**) Western blotting results of phosphorylated (p-)NF-κB and NF-κB protein expression. Full-length blots are shown in Supplementary Figure S1

The immortalized murine microglia cell line BV-2 was used as a substitute for primary microglia in the LPS-induced neuroinflammation model [25]. To exclude potential cytotoxicity at the experimental concentrations, we assessed BV-2 cell viability after roflumilast and zatolmilast exposure using the WST-8 assay (Fig. 1A). Nontoxic concentrations (≤ 10 µM) of roflumilast and zatolmilast were used in subsequent experiments.

Then we investigated whether these inhibited LPS-induced production of NO, an inflammatory mediator, and TNF-α, an inflammatory cytokine, in BV-2 cells. Compared to the untreated control, NO and TNF-α production were significantly increased after LPS treatment. Pretreatment with roflumilast showed a dose-dependent decrease in NO and TNF-α levels (Fig. 1B); however, zatolmilast did not have this effect (Fig. 1C). The calculated IC_50_ values of roflumilast against NO and TNF-α were 311.5 nM and 12.2 nM, respectively.

### Effects of roflumilast and zatolmilast on the NF-κB signaling pathway in LPS-exposed BV-2 cells

Crosstalk between PDE4 activity and the NF-κB pathway may play a major role in modulating inflammatory cytokine levels [1, 26]. To investigate the molecular mechanisms underlying the anti-inflammatory effects of the PDE4 inhibitors roflumilast and zatolmilast, we assessed their ability to inhibit NF-κB and p-NF-κB expression via Western blotting analysis (Fig. 1D). LPS treatment raised p-NF-κB levels compared to the control group. Roflumilast prevented the LPS-induced increase in p-NF-κB expression in a concentration-dependent manner, whereas zatolmilast did not.

### Effects of roflumilast and zatolmilast on inflammatory cytokine production in LPS-treated mice

**Fig. 2:**
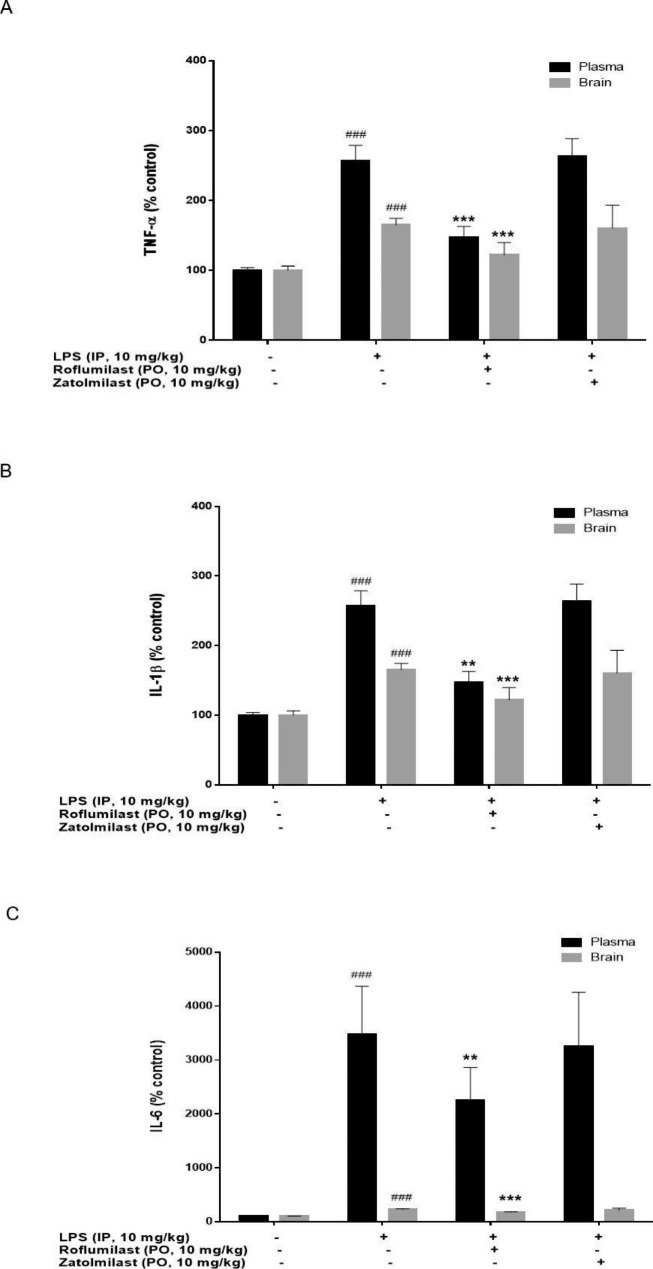
Anti-neuroinflammatory effects of roflumilast and zatolmilast in lipopolysaccharide (LPS)-treated mice. (**A**) Tumor necrosis factor-α (TNF-α), (**B**) interleukin-1β (IL-1β), and (**C**) IL-6 levels in plasma and brain homogenates of mice administered roflumilast and zatolmilast. C57BL/6J mice were administered roflumilast or zatolmilast (10 mg/kg) orally once daily for 4 consecutive days. Then, LPS (10 mg/kg) was injected intraperitoneally 2 h after the final drug administration. Mice were sacrificed and plasma and brain samples collected 8 h after the LPS injection. TNF-α, IL-1β, and IL-6 levels were determined by enzyme-linked immunosorbent assay. Data are expressed as the mean ± standard deviation (n = 7–9 per group). ^*###*^*P* < 0.001 versus the control group; ^**^P < 0.01, ^***^P < 0.001 versus the LPS group

Next, we assessed the effects of roflumilast and zatolmilast on LPS-induced neuroinflammation in vivo to validate the in vitro experiments (Fig. 2). Roflumilast and zatolmilast were administered to mice orally once daily for 4 consecutive days before intraperitoneal injection of LPS (10 mg/kg). Whereas LPS administration alone increased the levels of TNF-α, IL-1β, and IL-6 in both plasma and brain compared to the control, the levels in the roflumilast group were reduced. Zatolmilast did not reduce inflammatory cytokine production in vivo.

### Bidirectional MDR1-MDCK monolayer permeability of roflumilast and zatolmilast

Drug interactions with P-glycoprotein (P-gp), a major efflux transporter encoded by the *MDR1* gene, may be the primary mechanism hindering drug passage to the brain [27]. Therefore, we performed MDR1-MDCK monolayer permeability assays of roflumilast and zatolmilast (3, 10, and 30 µM) in both the apical-to-basolateral and basolateral-to-apical directions for 2 h. The P_app_ values of roflumilast and zatolmilast in the apical-to-basolateral direction were > 25.0 and 3.7 × 10^–6^ cm/s, respectively, and increased in a concentration-dependent manner (Table 1). The efflux ratios (ERs) of roflumilast and zatolmilast were < 0.95 and 1.92, respectively. According to FDA guidelines, P_app_ < 1 × 10^–6^ cm/s indicates poor permeability, P_app_ of 1–10 × 10^–6^ cm/s indicates moderate permeability, and P_app_ > 10 × 10^–6^ cm/s indicates high permeability. In addition, ER > 2 indicates the occurrence of drug efflux. Considering the FDA guidelines, roflumilast and zatolmilast exhibited high and moderately high permeability, respectively. These findings suggest that they are not P-gp substrates.

**Table 1:** Apparent permeability coefficients (P_app_) and efflux ratios for roflumilast and zatolmilast (3, 10, and 30 µM) using the MDR1-MDCK monolayer assay

Compound	Concentration (µM)	P_app_ (× 10^–6^ cm/s)		ER
		Apical → Basolateral	Basolateral → Apical	
Roflumilast	3	25.1 ± 1.28	23.9 ± 3.61	0.95
	10	44.5 ± 2.53 ^***^	40.8 ± 6.48 ^#^	0.91
	30	113 ± 5.70 ^***^	72.2 ± 6.32 ^###^	0.64
Zatolmilast	3	3.72 ± 0.33	7.18 ± 1.53	1.92
	10	9.59 ± 0.84 ^***^	18.0 ± 1.37 ^###^	1.89
	30	70.3 ± 9.58 ^***^	126 ± 8.39 ^###^	1.81

Data are expressed as the mean ± standard deviation, n = 3. ^***^P < 0.001 versus the apical-to-basolateral value at 3 µM; ^*#*^*P* < 0.05, ^*###*^*P* < 0.001 versus the basolateral-to-apical value at 3 µM

### Pharmacokinetics in mice

**Fig. 3:**
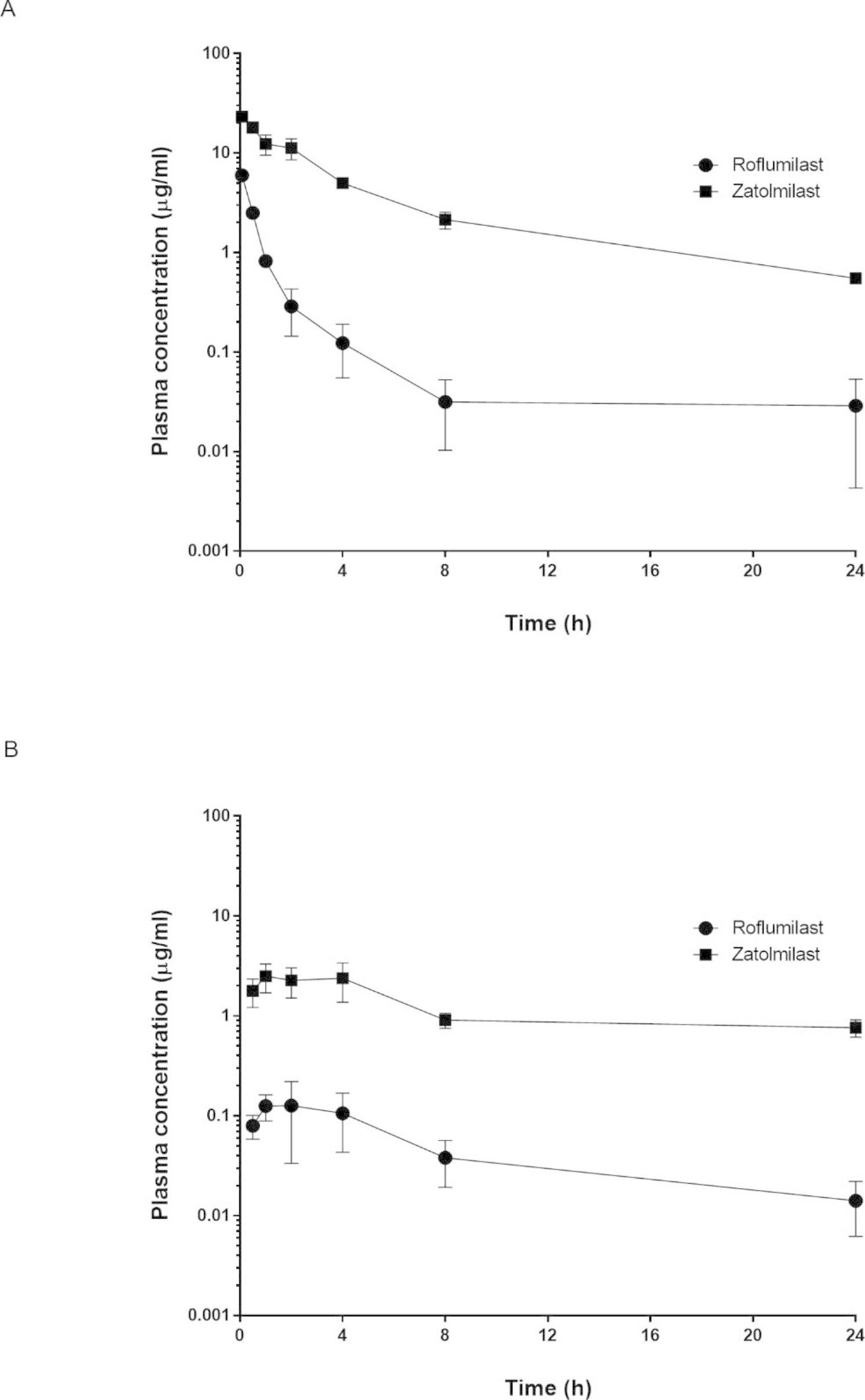
Plasma concentration–time curves of roflumilast and zatolmilast after intravenous and oral administration in mice. Data are expressed as the mean ± standard deviation (n = 3). (**A**) Intravenous administration (1 mg/kg); (**B**) Oral administration (10 mg/kg)

To clarify the pharmacokinetics of roflumilast and zatolmilast, we constructed mean plasma concentration–time curves and assessed non-compartmental pharmacokinetic parameters after intravenous and oral administration in mice. The results are presented in Fig. 3; Tables 2 and 3. After intravenous dosing (1 mg/kg), the AUCs of roflumilast and zatolmilast were 4.89 and 81.9 µg h/mL, respectively. These compounds had low systemic clearance and volume of distribution of 0.01–0.2 L/h/kg and 0.07–0.59 L/kg, respectively. At a dose of 10 mg/kg, roflumilast had a time-to-peak drug concentration (T_max_) of 2.33 h and peak concentration (C_max_) of 0.15 µg/mL. Zatolmilast had a T_max_ of 2 h and C_max_ of 2.53 µg/mL following oral administration. The oral bioavailabilities of roflumilast and zatolmilast were 2.3 and 3.7, respectively.

**Table 2:** Non-compartmental pharmacokinetic parameters of roflumilast and zatolmilast following intravenous administration (1 mg/kg) in mice

Parameter	Roflumilast	Zatolmilast
T_1/2_ (h)	3.74 ± 0.71	6.35 ± 0.62
AUC_last_ (µg·h/mL)	4.89 ± 0.94	81.88 ± 10.27
AUC_inf_ (µg·h/mL)	5.07 ± 1.10	86.94 ± 10.93
CL (L/h/kg)	0.20 ± 0.05	0.01 ± 0.002
V_ss_ (L/kg)	0.59 ± 0.21	0.07 ± 0.01

Data are expressed as the mean ± standard deviation, n = 3; T_1/2_, terminal half-life; AUC_last_, area under the plasma concentration–time curve from 0 to 24 h; AUC_inf_, area under the plasma concentration–time curve to infinity; CL, Clearance; V_ss_, volume of distribution at a steady state

**Table 3:** Non-compartmental pharmacokinetic parameters of roflumilast and zatolmilast following oral administration (10 mg/kg) in mice

Parameter	Roflumilast	Zatolmilast
T_max_ (h)	2.33 ± 1.53	2 ± 1.73
C_max_ (µg/mL)	0.15 ± 0.07	2.53 ± 0.86
T_1/2_ (h)	7.13 ± 2.86	20.37 ± 6.77
AUC_last_ (µg·h/mL)	1.13 ± 0.54	30.29 ± 9.64
AUC_inf_ (µg·h/mL)	1.29 ± 0.54	62.05 ± 31.8
F (%)	2.3	3.7

Data are expressed as the mean ± standard deviation, n = 3; T_max_, time to peak drug concentration; C_max_, peak drug concentration; T_1/2_, terminal half-life; AUC_last_, area under the plasma concentration–time curve from 0 to 24 h; AUC_inf_, area under the plasma concentration–time curve to infinity; F, bioavailability

### Brain disposition in mice

Finally, we evaluated the brain disposition of roflumilast and zatolmilast in mice. Plasma and brain tissue samples were obtained at 4 h after oral administration (10 mg/kg), and the drug concentrations were measured via LC-MS/MS. The results are presented in Table 4. The unbound plasma and brain concentrations were 0.39 ng/mL and 0.07 ng/g for roflumilast and 9.57 ng/mL and 1.7 ng/g for zatolmilast, respectively. The calculated K_p,uu_ values of roflumilast and zatolmilast were 0.17 and 0.18, respectively.

**Table 4:** Concentrations of roflumilast and zatolmilast in mouse plasma and brain tissues and the unbound brain-to-plasma partition coefficient (K_p,uu_) values following oral administration (10 mg/kg) in mice

Parameter		Roflumilast	Zatolmilast
Concentration (ng/mL or ng/g at 4 h)	Plasma	30.6 ± 14.2	3627 ± 890
	Brain	18.2 ± 12.9	1203 ± 345
Brain K_p_		0.53	0.33
F_u_ (%)	Plasma	1.26 ± 0.58	0.26 ± 0.01
	Brain	0.37 ± 0.20	0.14 ± 0.02
Brain K_p,uu_		0.17	0.18

Data are expressed as the mean ± standard deviation, n = 3. K_p_, brain-to-plasma ratio; F_u_, unbound drug fraction

## Discussion

We evaluated the in vitro and in vivo bioactivities of two selective PDE4 inhibitors, roflumilast and zatolmilast, against LPS-induced neuroinflammation, as well as their ability to cross the blood–brain barrier. Roflumilast, a PDE4 inhibitor, attenuated the production of NO and the inflammatory cytokines TNF-α, IL-1β, and IL-6 in both BV-2 microglial cells and C57BL/6J mice. However, zatolmilast, a PDE4D inhibitor, showed no anti-inflammatory effects. We further confirmed that the anti-inflammatory effects of roflumilast occurred via attenuation of p-NF-κB levels.

Barber et al. [26] reported that PDE4A4 was upregulated in lung macrophages of subjects with COPD; this is thought to play a major role in the pathogenesis of COPD. This suggests that PDE4A4 might be a PDE4 isoform-specific therapeutic target for COPD. On the other hand, analysis of mRNA levels reveals that PDE4B and PDE4D are the most abundant subfamilies throughout the human, monkey, and rat brains [28]. In addition, TNF-α increases PDE4B expression and nuclear translocation in microglia and PDE4B is highly expressed in activated microglia after traumatic brain injury and spinal cord injury [29]. Thus, it is reasonable to believe that roflumilast may have anti-neuroinflammatory activity primarily by inhibiting PDE4B in BV-2 cells and mice, unlike the anti-inflammatory effect of PDE4A inhibition in COPD.

A crucial mechanism of LPS-induced neuroinflammation is the toll-like receptor 4 (TLR4) signaling pathway. In the response to LPS, TLR4 is an important mediator of several inflammatory pathways and binds to MyD88 to activate NF-κB [30]. It is mainly expressed in microglia of the CNS, and abnormal expression causes CNS injury, including the degeneration of neurons, synaptic loss, and neuronal cell death [23, 31]. This process is mediated by the production of inflammatory cytokines such as TNF-α, IL-1β, and IL-6, increased activity of iNOS, COX-2, β-secretase, and γ-secretase, Aβ accumulation, and oxidative stress [32, 33]. Therefore, LPS can induce neuroinflammation and amyloidogenesis in neurons [34, 35].

The PDE4–cAMP–protein kinase A (PKA)–CREB–NF-κB signaling pathway is closely related to inflammatory responses. PDE4 inhibition increases cAMP levels, in turn activating PKA. PKA activity modulates CREB phosphorylation and NF-κB transcription [5]. LPS injections increase neuroinflammation and damages the BBB [36], where PDE4B is the main PDE4 subfamily engaged in the LPS response [37, 38]. By contrast, TLR ligation has no effect on *PDE4D* gene expression [7]. Thus, PDE4B inhibition, but not PDE4D, may be necessary for the treatment of TLR-dependent neuroinflammation.

To assess the PDE4 inhibitors roflumilast and zatolmilast for use for the treatment of CNS disorders, we assessed MDR1-MDCK monolayer permeability and mouse brain disposition. To treat brain diseases effectively, a drug must penetrate the BBB [39]. Therefore, identifying the disposition of drugs in the brain is an important step in CNS drug development. In the pharmaceutical industry, K_p,uu,brain_ is often used in pharmacokinetic–pharmacodynamic assessments of biological effects on the brain and to predict an effective dose [40]. In the present study, both roflumilast and zatolmilast had ERs < 2.0 in the MDR1-MDCK monolayer, indicating that they are not substrates of P-gp. In vivo, both compounds had long T_max_ values of 2–4 h and long terminal half-lives > 7 h after oral dosing (10 mg/kg). Plasma concentrations of roflumilast at all times after oral dosing were above the IC_50_ value against TNF-α (12.2 nM; 4.9 ng/mL), correlating with reduced TNF- α levels in roflumilast-treated mice.

Compounds with K_p,uu,brain_ > 0.3–0.5 can penetrate the BBB [41]. Their K_p,uu,brain_ values < 0.18 suggest poor CNS penetration by both roflumilast and zatolmilast. Although the unbound brain concentrations of roflumilast and zatolmilast at T_max_ were 0.07 ng/g (0.17 nM) and 1.7 ng/g (4.2 nM), respectively, the value of roflumilast was similar to the PDE4B inhibitory concentration (0.2 nM). The treatment of inflammatory diseases by PDE4 inhibitors such as rolipram and roflumilast has been limited due to side effects such as emesis and vomiting [42, 43]. The calculated oral emetic dosages of roflumilast in mice and rats are 9–30 mg/kg [9]. Robichaud et al. reported that PDE4D, but not PDE4B, is responsible for emesis and other side effects [43]. Selective PDE4B inhibitors have anti-inflammatory effects without side effects [43]. This evidence makes it reasonable to believe that inhibitors with higher selectivity for PDE4B vs. PDE4D should have fewer side effects.

## Conclusions

This study demonstrates differential pharmacological effects of two PDE4 inhibitors in both in vitro and in vivo LPS-induced neuroinflammation models. The findings suggest selective PDE4B inhibition as a potential therapeutic target for neuroinflammatory diseases.

## Methods

### Chemicals and reagents

Roflumilast and zatolmilast were purchased from MedChemExpress (Monmouth Junction, NJ, USA). LPS from *Escherichia coli* (0111:B4), Griess reagent, and disopyramide were purchased from Sigma Aldrich (St. Louis, MO, USA). Mouse TNF-α, IL-1β, and IL-6 enzyme-linked immunosorbent assay (ELISA) kits were purchased from R&D Systems (Minnesota, MN, USA). High-performance liquid chromatography (HPLC)-grade solvents were used for HPLC tandem mass spectrometry (MS/MS) analysis. The other chemicals and reagents were of the highest grade available.

### Cell culture

BV-2 cells, a murine microglial cell line, were purchased from AcceGen (Fairfield, NJ, USA) and cultured in Dulbecco’s Modified Eagle’s Medium (DMEM) (Corning, Riverfront, NY, USA) supplemented with 10% fetal bovine serum (FBS), 100 U/mL penicillin, and 100 µg/mL streptomycin. MDR1-MDCK cells, a Madin–Darby canine kidney cell line, were obtained from Dr. Piet Borst (Netherlands Cancer Institute, Amsterdam, the Netherlands) and cultured in DMEM containing 10% FBS, 100 U/mL penicillin, and 100 µg/mL streptomycin. Cells were incubated with 5% CO_2_ and an atmosphere of 95% humidity at 37℃.

### Cell viability assay

Cell viability was assessed using the WST-8 assay with Cyto X reagent (LPS Solution, Daejeon, Korea). BV-2 microglial cells were seeded at a density of 5 × 10^5^ cells/mL in 96-well plates. After incubation for 24 h, the cells were treated with 0–100 µM of either roflumilast or zatolmilast and exposed for 24 h. Finally, Cyto X solution was loaded in each well and incubated for 20 min. Absorbance was measured at 450 nm using a spectrophotometer (BioTek, Winooski, VT, USA).

### In vitro LPS-induced neuroinflammation model

BV-2 cells were seeded at a density of 5 × 10^5^ cells/mL in 96-well plates. After 24 h, the cell culture medium was replaced with DMEM containing 1% FBS, and cells were treated with various concentrations of roflumilast and zatolmilast for 1 h. Finally, 1 µg/mL LPS was added to the cell culture medium for 24 h to induce inflammation.

### In vivo LPS-induced neuroinflammation mouse model

Eight-week-old male C57BL/6J mice (body weight 22–26 g) were purchased from Orient Bio Inc. (Seongnam, Korea). The animals were acclimatized to a temperature-controlled room at 23℃ with a 12 h light/12 h dark cycle for 7 days before the experiment; the mice received water and food *ad libitum*. All procedures complied with the Guidelines for the Care and Use of Laboratory Animals of the Animal Ethics Committee of the Korea Research Institute of Chemical Technology.

To assess anti-neuroinflammatory effects of roflumilast and zatolmilast in vivo, C57BL/6J mice were divided into four groups (n = 7–9 in each group): control group, LPS group, LPS + roflumilast 10 mg/kg group, and LPS + zatolmilast 10 mg/kg group. The tested compounds were solved in the vehicle (10% N-methyl-2-pyrrolidone, 60% polyethylene glycol 400, 30% distilled water) immediately before the experiment. The dosing volume was 5 mL/kg. In the treatment groups, roflumilast and zatolmilast were administered orally once daily for 4 consecutive days. On day 4, all groups (except the control) received an intraperitoneal injection of LPS (10 mg/kg) 2 h after drug administration. Finally, 8 h after LPS injection, the mice were sacrificed via CO_2_ inhalation. Plasma and brain samples were collected and stored at − 80℃ until analysis.

### Measurement of NO

Frozen brain tissues in cold 0.1 M phosphate-buffered saline were homogenized using the FastPrep-24™ Classic homogenizer (MP Biomedicals, Irvine, California, USA). The homogenates were centrifuged at 15,000 rpm for 10 min, and the supernatants were collected. To assess NO levels, the cell culture supernatant and brain tissue pellet samples were reacted with an equal volume of Griess reagent at room temperature for 10 min. Absorbance was measured at 540 nm using a spectrophotometer (BioTek).

### TNF-α, IL-1β, and IL-6 ELISA

To measure TNF-α, IL-1β, and IL-6 levels in each sample, ELISA was performed using mouse TNF-α, IL-1β, and IL-6 ELISA kits (R&D Systems). The absorbance was measured at 450 nm using a spectrophotometer (BioTek).

### Western blotting analysis

BV-2 cells were treated with roflumilast (1 and 10 µM), zatolmilast (1 and 10 µM), or 0.5% dimethyl sulfoxide for 1 h. Then the cells were incubated with or without LPS (1 µg/mL) for 4 h. Cells were harvested using trypsin and washed twice in cold Dulbecco’s phosphate-buffered saline and lysed with RIPA lysis buffer (Elpis, Daejeon, Korea) containing protease and phosphatase inhibitor mini tablets (Thermo Fisher, Waltham, MA, USA). Protein lysate concentrations were determined using the Pierce BCA Protein Assay kit (Thermo Fisher). Protein samples (5 µg) were separated via 5–20% sodium dodecyl sulfate polyacrylamide gel electrophoresis and then transferred to polyvinylidene fluoride membranes (ATTO, Tokyo, Japan). After transfer, membranes were blocked using Tris-buffered saline containing 0.5% Tween 20 and 5% bovine serum albumin at room temperature for 1 h. The membranes were incubated overnight at 4℃ with primary antibodies at a dilution ratio of 1:1000. NF-κB (cat no. #8242, Cell Signaling Technology, MA, USA), phosphorylated (p-)NF-κB (cat no. #3033, Cell Signaling Technology), and GAPDH (cat no. #5174, Cell Signaling Technology) were used as primary antibodies. Subsequently, blots were detected using horseradish peroxidase-conjugated secondary antibodies (1:10,000 dilution) and the ECL substrate (Millipore Corporation, Billerica, MA, USA) at room temperature for 1 h. Protein expression levels were measured using WSE-6100 LuminoGraph (ATTO). The bands were analyzed using the ImageJ software (NIH, Bethesda, MD, USA).

### LC-MS/MS analysis

LC-MS/MS analyses were performed on an Agilent 1200 Series HPLC System (Agilent Technology, Palo Alto, CA, USA) coupled to an API 4000 Qtrap mass spectrometer equipped with an electrospray-ionization source operated in positive-ionization mode (AB SCIEX, Foster City, CA, USA). Multiple reaction monitoring (MRM) mode was used for targeted analysis. The optimized instrument conditions were as follows: curtain gas, 10 psi; ion spray voltage, 5500 V; temperature, 550℃; nebulizing gas (GS1), 50 psi; heating gas (GS2), 50 psi. MRM was *m*/*z* 403.1 → 167.1 for roflumilast, 406.1 → 290.1 for zatolmilast, and 340.1 → 239.2 for disopyramide (internal standard). All data were measured using Analyst Software version 1.4.2 (AB SCIEX).

The LC chromatograph was equipped with a Unison UK-C18 column (100 × 2.0 mm i.d., 3 μm; Imtakt, Portland, Oregon, USA). The mobile phase consisted of 0.1% formic acid in water (A) and acetonitrile (B), and was filtered and degassed before use. Roflumilast and zatolmilast were analyzed in isocratic elution mode or gradient elution mode. The isocratic elution mode conditions were 0–2.5 min, 80% B; the flow rate was 0.3 mL/min. The gradient elution mode conditions were 0–0.2 min, 5% B; 0.2–2.3 min, linear increase to 95% B; 2.3–2.4 min, 95% B; 2.4–2.5 min, linear decrease to 5% B; 2.5–5 min, 5% B; the flow rate was 0.35 mL/min.

### MDR1-MDCK monolayer permeability analysis

MDR1-MDCK cells were seeded at a density of 2 × 10^5^ cells/well on the apical side of 24-transwell plates with a pore size of 0.4 μm (Corning Costar, Cambridge, MA, USA). Each basolateral chamber was filled with 0.6 mL medium, and each apical side with 0.2 mL medium containing cells. The plates seeded with cells were incubated for 3 days. The culture medium was replaced on both the apical and basolateral sides 2 days after seeding. Before the experiment, the transepithelial electrical resistance was measured using an epithelial voltohmmeter (Millicell ERS-2; Millipore), where 300 ohms or greater was considered to indicate the formation of a cell monolayer with tight junctions. The cells were rinsed twice with pre-warmed Hank’s balanced salt solution (HBSS) containing 10 mM of HEPES and were incubated in a CO_2_ incubator for 30 min. Before the transport assay, roflumilast, zatolmilast, and reference compounds (atenolol, propranolol, and loperamide) were diluted with HBSS containing 10 mM HEPES to prepare the desired concentrations. The final concentrations of roflumilast and zatolmilast were 3, 10, and 30 µM. After equilibration, the solutions containing the test or reference compounds were added to either the apical side (0.2 mL) or basolateral side (0.6 mL) of the wells, and the plates were incubated in a shaking incubator (37℃, 200 rpm). Samples were collected every 30 min. To test for apical-to-basolateral flow, 110 µL sample was removed from each basolateral chamber; to test for basolateral-to-apical flow, 110 µL sample was taken from each apical chamber; then, each well was replenished with 110 µL fresh HBSS. To each sample, 50 µL internal standard (5 ng/mL disopyramide) in water was added. Samples were prepared for analysis using solid-phase extraction, and the sample concentrations were analyzed via LC-MS/MS. The permeability of the tested compounds was evaluated by calculating the apparent permeability coefficient (P_app_, cm/s) and the efflux ratio (ER). The P_app_ was calculated as follows:

$${\text{P}}_{app}=\frac{\text{d}\text{Q}}{\text{d}\text{t}}\times \frac{1}{\text{A}}\times \frac{1}{{\text{C}}_{0}}$$

where dQ/dt is the initial permeability rate of the compound across the cells (ng/s), A is the surface area of the cell monolayer (cm^2^), and C_0_ is the initial concentration of the donor side (ng/mL).

From the apical-to-basolateral and basolateral-to-apical P_app_ values, the ER was calculated as follows:

$${\rm{Efflux}}\,{\rm{Ratio}} = \frac{{{{\rm{P}}_{app\,\,\left( {Basolateral\, \to \,Apical} \right)}}}}{{{{\rm{P}}_{app\,\,\left( {Apical\, \to \,Basolateral} \right)}}}}$$

### In vitro protein-binding assay using equilibrium dialysis

The roflumilast and zatolmilast solutions were prepared to a final concentration of 5 µM by spiking 6 µL 500 µM stock solution in 594 µL pooled mouse plasma and mouse blank brain homogenate. A rapid equilibrium dialysis device with inserts (cat no. #90,112, Thermo Fisher) was used for the protein-binding assay. First, 100 µL roflumilast or zatolmilast solution was loaded into the red chamber and 350 µL prepared BupH™ phosphate-buffered saline (cat no. #28,372, Thermo Fisher) was loaded into the white chamber. The samples were incubated for 4 h in a shaking incubator (37℃, 300 rpm). After incubation, 50 µL buffer from the white chamber was added to 50 µL fresh mouse plasma or brain homogenate, and 50 µL dosing solution from the red chamber was added to 50 µL fresh buffer to adjust for matrix effects. To each sample, 200 µL ice-cold acetonitrile with internal standard was added. The samples were centrifuged at 4,000 rpm for 20 min and the supernatants were collected for LC-MS/MS analysis. The unbound fraction of drug (F_u_) in plasma and brain tissue was calculated as follows based on previous studies [45, 46]:

$${{\text{F}}_{{\text{u,plasma}}\,{\text{or}}\,{\text{dh}}}}\,\left( \% \right) = \frac{{{C_{protein-free\,compartment}}}}{{{C_{protein-containing\,compartment}}}} \times 100$$

$${{\text{F}}_{{\text{u,}}\,{\text{brain}}}}\,\left( \% \right) = \frac{{1/D}}{{\left[ {\left( {\frac{1}{{Fu,\,dh}}} \right) - 1} \right] + \left( {\frac{1}{D}} \right)}}$$

where *C*_*protein-free compartment*_ and *C*_*protein-containing compartment,*_ D, and dh are the concentrations of drug in the buffer chamber and drug solution chamber, dilution factor, and diluted brain homogenate, respectively.

The values were calculated using the measured diluted free fractions and the respective dilution factor.

### Pharmacokinetics and brain disposition in mice

Male C57BL/6J mice (aged 8 weeks, 22–26 g) were purchased from Orient Bio and used to assess the pharmacokinetics of roflumilast and zatolmilast. All mice were acclimated to a temperature-controlled room at 23℃ for 7 days and fasted for 12 h before the experiment.

For the pharmacokinetic study, mice were administrated roflumilast or zatolmilast intravenously and orally. Blood samples were collected at 0.083, 0.5, 1, 2, 4, 8, and 24 h after drug administration. Plasma samples were obtained via centrifugation at 15,000 rpm for 3 min and stored at − 20℃ until LC-MS/MS analysis.

For the brain disposition evaluation, 4 h after oral administration (10 mg/kg), the mice were sacrificed via CO_2_ inhalation. Residual blood in the brain was removed from circulation via cardiac perfusion with saline solution. Blood and brain samples were collected and stored at − 20℃ until LC-MS/MS analysis.

Plasma and brain standard calibration curves were prepared to final concentrations of 1.95–8000 ng/mL. For both plasma and brain samples, 30 µL each sample was vortexed with 270 µL acetonitrile containing disopyramide as the internal standard for protein precipitation. The samples were centrifuged at 15,000 rpm for 10 min and the supernatants were analyzed by LC-MS/MS. The brain-to-plasma ratio (K_p_) and unbound K_p_ (K_p,uu_) were calculated as follows:

$${\text{K}}_{\text{P}}= {\text{C}}_{\text{b}\text{r}\text{a}\text{i}\text{n}}/{\text{C}}_{\text{p}\text{l}\text{a}\text{s}\text{m}\text{a}}$$

$${\text{K}}_{\text{p},\text{u}\text{u}}={\text{K}}_{\text{p}} \times \frac{{\text{f}\text{u}}_{\text{b}\text{r}\text{a}\text{i}\text{n}}}{{\text{f}\text{u}}_{\text{p}\text{l}\text{a}\text{s}\text{m}\text{a}}}$$

### Pharmacokinetic analysis

Pharmacokinetic parameters were calculated via non-compartmental analysis using Phoenix WinNonlin, ver. 8.3 (Pharsight, Mountain View, CA, USA). The area under the plasma concentration–time curve from 0 to 24 h (AUC_last_) and infinity (AUC_inf_) were determined following the linear trapezoidal rule.

### Statistical analysis

All experimental data were collected in triplicate and are expressed as the mean ± standard deviation. Statistical analyses were evaluated using one-way analysis of variance followed by Tukey’s *post hoc* test with GraphPad Prism 9.4 (GraphPad Software, San Diego, CA, USA). Significant differences were defined as *p* values < 0.05, < 0.01, or < 0.001.

## Electronic supplementary material

Below is the link to the electronic supplementary material.

Additional file 1: Supplementary Fig. 1D. Western blotting results of p-NF-κB and NF-κB protein expression.

## Data Availability

The data presented in this study are available on request from the corresponding author.

## References

[1] Tibbo AJ, Baillie GS. Phosphodiesterase 4B: Master Regulator of Brain Signaling. Cells 2020, 9(5). 10.3390/cells9051254 PMC7291338 32438615

[2] Conti M, Beavo J (2007). Biochemistry and physiology of cyclic nucleotide phosphodiesterases: essential components in cyclic nucleotide signaling. Annu Rev Biochem.

[3] Lugnier C (2006). Cyclic nucleotide phosphodiesterase (PDE) superfamily: a new target for the development of specific therapeutic agents. Pharmacol Ther.

[4] Lugnier C (2022). The complexity and multiplicity of the specific cAMP phosphodiesterase family: PDE4, open New adapted therapeutic approaches. Int J Mol Sci.

[5] Pearse DD, Hughes ZA (2016). PDE4B as a microglia target to reduce neuroinflammation. Glia.

[6] Pearse D, Jarnagin K (2010). Abating progressive tissue injury and preserving function after CNS trauma: the role of inflammation modulatory therapies. Curr Opin Investig Drugs.

[7] Borysiewicz E, Fil D, Dlaboga D, O’Donnell JM, Konat GW (2009). Phosphodiesterase 4B2 gene is an effector of toll-like receptor signaling in astrocytes. Metab Brain Dis.

[8] Hatzelmann A, Morcillo EJ, Lungarella G, Adnot S, Sanjar S, Beume R, Schudt C, Tenor H (2010). The preclinical pharmacology of roflumilast–a selective, oral phosphodiesterase 4 inhibitor in development for chronic obstructive pulmonary disease. Pulm Pharmacol Ther.

[9] Wu Q, Qi L, Li H, Mao L, Yang M, Xie R, Yang X, Wang J, Zhang Z, Kong J (2017). Roflumilast reduces cerebral inflammation in a rat model of experimental subarachnoid hemorrhage. Inflammation.

[10] Vanmierlo T, Creemers P, Akkerman S, van Duinen M, Sambeth A, De Vry J, Uz T, Blokland A, Prickaerts J (2016). The PDE4 inhibitor roflumilast improves memory in rodents at non-emetic doses. Behav Brain Res.

[11] Feng H, Wang C, He W, Wu X, Li S, Zeng Z, Wei M, He B (2019). Roflumilast ameliorates cognitive impairment in APP/PS1 mice via cAMP/CREB/BDNF signaling and anti-neuroinflammatory effects. Metab Brain Dis.

[12] Wu Q, Qi L, Li H, Mao L, Yang M, Xie R, Yang X, Wang J, Zhang Z, Kong J (2017). Roflumilast reduces cerebral inflammation in a rat model of experimental subarachnoid hemorrhage. Inflammation.

[13] Wang Y, Gao S, Zheng V, Chen L, Ma M, Shen S, Qu J, Zhang H, Gurney ME, O’Donnell JM (2020). A novel PDE4D inhibitor BPN14770 reverses scopolamine-induced cognitive deficits via cAMP/SIRT1/Akt/Bcl-2 pathway. Front cell Dev biology.

[14] Gurney ME, Nugent RA, Mo X, Sindac JA, Hagen TJ, Fox IIID, O’Donnell JM, Zhang C, Xu Y, Zhang H-T. Design and synthesis of selective phosphodiesterase 4D (PDE4D) allosteric inhibitors for the treatment of fragile X syndrome and other brain disorders. In.: ACS Publications; 2019. 10.1021/acs.jmedchem.9b00193 PMC7444661 31013090

[15] Berry-Kravis EM, Harnett MD, Reines SA, Reese MA, Ethridge LE, Outterson AH, Michalak C, Furman J, Gurney ME (2021). Inhibition of phosphodiesterase-4D in adults with fragile X syndrome: a randomized, placebo-controlled, phase 2 clinical trial. Nat Med.

[16] Prickaerts J, Heckman PR, Blokland A (2017). Investigational phosphodiesterase inhibitors in phase I and phase II clinical trials for Alzheimer’s disease. Expert Opin Investig Drugs.

[17] Wang Y, Gao S, Zheng V, Chen L, Ma M, Shen S, Qu J, Zhang H, Gurney ME, O’Donnell JM (2020). A novel PDE4D inhibitor BPN14770 reverses Scopolamine-Induced Cognitive Deficits via cAMP/SIRT1/Akt/Bcl-2 pathway. Front Cell Dev Biol.

[18] Prinz M, Priller J (2014). Microglia and brain macrophages in the molecular age: from origin to neuropsychiatric disease. Nat Rev Neurosci.

[19] Gee MS, Kim SW, Kim N, Lee SJ, Oh MS, Jin HK, Bae JS, Inn KS, Kim NJ, Lee JK (2018). A novel and selective p38 mitogen-activated protein kinase inhibitor attenuates LPS-Induced Neuroinflammation in BV2 Microglia and a mouse model. Neurochem Res.

[20] Cunningham CL, Martínez-Cerdeño V, Noctor SC (2013). Microglia regulate the number of neural precursor cells in the developing cerebral cortex. J Neurosci.

[21] Ueno M, Fujita Y, Tanaka T, Nakamura Y, Kikuta J, Ishii M, Yamashita T (2013). Layer V cortical neurons require microglial support for survival during postnatal development. Nat Neurosci.

[22] Shao F, Wang X, Wu H, Wu Q, Zhang J. Microglia and neuroinflammation: crucial pathological mechanisms in traumatic brain injury-induced neurodegeneration. Front Aging Neurosci 2022, 14. 10.3389/fnagi.2022.825086 PMC8990307 35401152

[23] Nam HY, Nam JH, Yoon G, Lee JY, Nam Y, Kang HJ, Cho HJ, Kim J, Hoe HS (2018). Ibrutinib suppresses LPS-induced neuroinflammatory responses in BV2 microglial cells and wild-type mice. J Neuroinflammation.

[24] Konsman JP. Cytokines in the brain and neuroinflammation: we didn’t starve the fire! Pharmaceuticals 2022, 15(2):140. 10.3390/ph15020140 PMC8878213 35215252

[25] Henn A, Lund S, Hedtjärn M, Schrattenholz A, Pörzgen P, Leist M (2009). The suitability of BV2 cells as alternative model system for primary microglia cultures or for animal experiments examining brain inflammation. ALTEX: Altern Anim experimentation.

[26] Barber R, Baillie GS, Bergmann R, Shepherd MC, Sepper R, Houslay MD, Heeke GV (2004). Differential expression of PDE4 cAMP phosphodiesterase isoforms in inflammatory cells of smokers with COPD, smokers without COPD, and nonsmokers. Am J Physiology-Lung Cell Mol Physiol.

[27] Wang Q, Rager JD, Weinstein K, Kardos PS, Dobson GL, Li J, Hidalgo IJ (2005). Evaluation of the MDR-MDCK cell line as a permeability screen for the blood-brain barrier. Int J Pharm.

[28] Pérez-Torres S, Miró X, Palacios JM, Cortés R, Puigdoménech P, Mengod G (2000). Phosphodiesterase type 4 isozymes expression in human brain examined by in situ hybridization histochemistry and [3H] rolipram binding autoradiography: comparison with monkey and rat brain. J Chem Neuroanat.

[29] Ghosh M, Xu Y, Pearse DD (2016). Cyclic AMP is a key regulator of M1 to M2a phenotypic conversion of microglia in the presence of Th2 cytokines. J Neuroinflamm.

[30] Nair AR, Masson GS, Ebenezer PJ, Del Piero F, Francis J (2014). Role of TLR4 in lipopolysaccharide-induced acute kidney injury: protection by blueberry. Free Radic Biol Med.

[31] Zhao J, Bi W, Xiao S, Lan X, Cheng X, Zhang J, Lu D, Wei W, Wang Y, Li H (2019). Neuroinflammation induced by lipopolysaccharide causes cognitive impairment in mice. Sci Rep.

[32] Yang L, Zhou R, Tong Y, Chen P, Shen Y, Miao S, Liu X (2020). Neuroprotection by dihydrotestosterone in LPS-induced neuroinflammation. Neurobiol Dis.

[33] Kshirsagar V, Thingore C, Gursahani M, Gawali N, Juvekar A (2021). Hydrogen sulfide ameliorates lipopolysaccharide-induced memory impairment in mice by reducing apoptosis, oxidative, and inflammatory effects. Neurotox Res.

[34] Gu SM, Lee HP, Ham YW, Son DJ, Kim HY, Oh KW, Han S-B, Yun J, Hong JT (2018). Piperlongumine improves lipopolysaccharide-induced amyloidogenesis by suppressing NF-KappaB pathway. Neuromol Med.

[35] Lee JW, Lee YK, Yuk DY, Choi DY, Ban SB, Oh KW, Hong JT (2008). Neuro-inflammation induced by lipopolysaccharide causes cognitive impairment through enhancement of beta-amyloid generation. J Neuroinflamm.

[36] Noh H, Jeon J, Seo H (2014). Systemic injection of LPS induces region-specific neuroinflammation and mitochondrial dysfunction in normal mouse brain. Neurochem Int.

[37] Ghosh M, Garcia-Castillo D, Aguirre V, Golshani R, Atkins CM, Bramlett HM, Dietrich WD, Pearse DD (2012). Proinflammatory cytokine regulation of cyclic AMP‐phosphodiesterase 4 signaling in microglia in vitro and following CNS injury. Glia.

[38] Jin S-LC, Lan L, Zoudilova M, Conti M (2005). Specific role of phosphodiesterase 4B in lipopolysaccharide-induced signaling in mouse macrophages. J Immunol.

[39] Lee JH, Ji SH, Lim JS, Ahn S, Yun H-y, Kim SH, Song JS. Anti-neuroinflammatory Effects and Brain Pharmacokinetic Properties of Selonsertib, an apoptosis signal-regulating kinase 1 inhibitor, in mice. Neurochem Res 2022:1–9. 10.1007/s11064-022-03777-9 36309631

[40] Loryan I, Reichel A, Feng B, Bundgaard C, Shaffer C, Kalvass C, Bednarczyk D, Morrison D, Lesuisse D, Hoppe E. Unbound brain-to-plasma partition coefficient, kp, uu, brain—a game changing parameter for CNS drug Discovery and Development. Pharm Res 2022:1–21. 10.1007/s11095-022-03246-6 PMC9246790 35411506

[41] Kalvass JC, Olson ER, Cassidy MP, Selley DE, Pollack GM (2007). Pharmacokinetics and pharmacodynamics of seven opioids in P-glycoprotein-competent mice: assessment of unbound brain EC50, u and correlation of in vitro, preclinical, and clinical data. J Pharmacol Exp Ther.

[42] Bateman ED, Izquierdo JL, Harnest U, Hofbauer P, Magyar P, Schmid-Wirlitsch C, Leichtl S, Bredenbröker D (2006). Efficacy and safety of roflumilast in the treatment of asthma. Ann Allergy Asthma Immunol.

[43] Robichaud A, Stamatiou PB, Jin S-LC, Lachance N, MacDonald D, Laliberté F, Liu S, Huang Z, Conti M, Chan C-C (2002). Deletion of phosphodiesterase 4D in mice shortens α 2-adrenoceptor–mediated anesthesia, a behavioral correlate of emesis. J Clin Investig.

[44] Naganuma K, Omura A, Maekawara N, Saitoh M, Ohkawa N, Kubota T, Nagumo H, Kodama T, Takemura M, Ohtsuka Y (2009). Discovery of selective PDE4B inhibitors. Bioorg Med Chem Lett.

[45] Kariv I, Cao H, Oldenburg KR (2001). Development of a high throughput equilibrium dialysis method. J Pharm Sci.

[46] Reichel A (2009). Addressing central nervous system (CNS) penetration in drug discovery: basics and implications of the evolving new concept. Chem Biodivers.

