# Supplementary material for: Differential effects of two phosphodiesterase 4 inhibitors against lipopolysaccharide-induced neuroinflammation in mice
Source: BMC Neurosci. 2023 Jul 31;24:39. doi: 10.1186/s12868-023-00810-7 (PMC10391911; doi:10.1186/s12868-023-00810-7)

Additional File 1

Note: All band examples from manuscript figures are denoted with a red box

P-NF-κB (65 kDa)

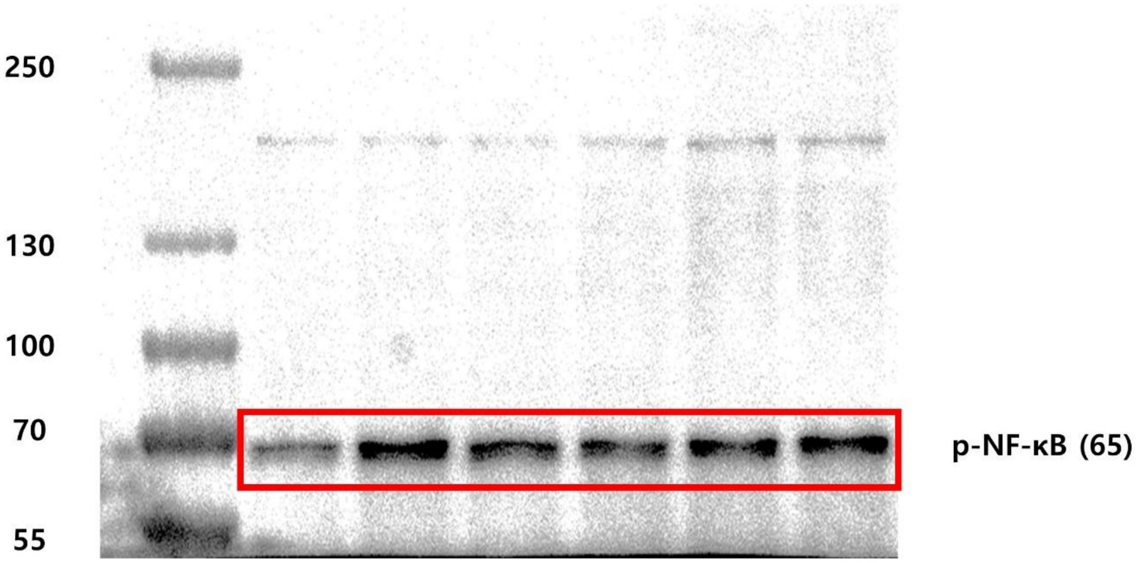

NF-κB (65 kDa)

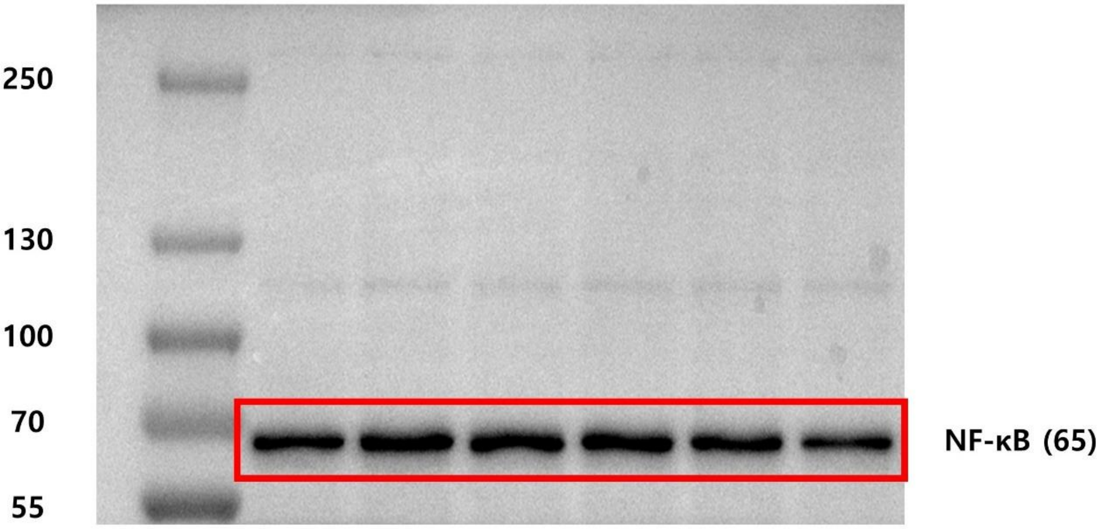

GAPDH (37 kDa)

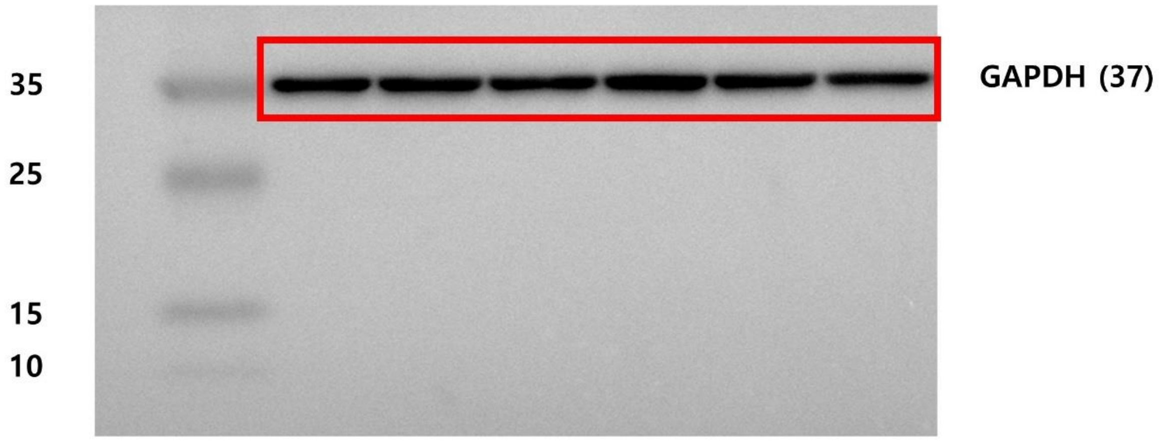

Supplement: Supplementary file 1 — Additional file 1: Supplementary Fig. 1D. Western blotting results of p-NF-κB and NF-κB protein expression. [file 12868_2023_810_MOESM1_ESM.pdf]
